# Adverse effects of pre-pregnancy maternal underweight on pregnancy and perinatal outcomes in a freeze-all policy

**DOI:** 10.1186/s12884-020-03509-3

**Published:** 2021-01-07

**Authors:** Shengluan Tang, Jialyu Huang, Jiaying Lin, Yanping Kuang

**Affiliations:** Department of Assisted Reproduction, Shanghai Ninth People’s Hospital, Shanghai Jiao Tong University School of Medicine, 639 Zhizaoju Rd, Shanghai, 200011 China

**Keywords:** Underweight, Overweight, Frozen embryo transfer, Pregnancy outcomes, Perinatal outcomes

## Abstract

**Background:**

Underweight and overweight may affect reproduction and interfere with treatment of infertility. In the present retrospective analysis, we sought to evaluate the effect of low body mass index (BMI) on pregnancy and perinatal outcomes in frozen–thawed embryo transfer (FET) cycles.

**Methods:**

This study involved 8755 FET cycles in a single IVF center during the period from January 2009 to December 2018. Both pregnancy and perinatal outcomes were assessed in women who were underweight, normal weight, and overweight as defined based on a respective BMI < 18.5 kg/m^2^, ≥ 18.5 BMI < 24.9 kg/m^2^, and BMI ≥ 25 kg/m^2^.

**Results:**

Being underweight was linked to reduced implantation rates as compared to a normal weight (33.56% vs. 37.26%). Similarly, when comparing outcomes in underweight women to those in normal weight women, rates of clinical pregnancy (48.14% vs. 53.85%) and ongoing pregnancy (43.04% vs. 50.47%) were reduced. Rates of miscarriage were markedly reduced in the normal weight group relative to the overweight group (10.73% vs. 13.37%). Perinatal outcomes were largely comparable for all groups, with the exception of very low birth weight rates (normal weight:0.58% vs. overweight: 2.03%), very small for gestational age rates (normal weight:1.31% vs. overweight:3.55%) and very preterm delivery rates (normal weight:0.82% vs. overweight: 2.03%), which were significantly elevated for overweight mothers.

**Conclusions:**

These results indicate that being underweight is linked to negative pregnancy outcomes when undergoing FET-based IVF.

## Background

Reproductive outcomes have been shown to be linked to maternal weight in a wide variety of studies. For example, women who are either underweight or overweight/obese have higher rates of anovulatory infertility [1]. Indeed, there are many studies indicating that obesity adversely affects fertility [2, 3], the effectiveness of assisted reproductive technology (ART) [4, 5], and pregnancy/obstetric outcomes [6, 7]. There is, however, very little data with respect to how being underweight (body mass index [BMI] < 18.5 kg/m^2^) influences these same outcomes. Studies that have been published provide inconsistent findings, potentially as a consequence of variability in methodology, inclusion criteria, BMI definitions, outcomes assessed, or adjustments based on confounding variables [8]. In addition, while some studies of IVF report on multiple treatment outcomes, some report only upon the first treatment cycle so as to prevent analytical complications stemming from the analysis of multiple, non-independent cycles in a given individual [9].

Frozen-embryo transfer is a technique that is now being widely applied, with more and more frozen cycles being employed worldwide [10]. This strategy depends upon the initial cryopreservation of all embryos, which are, upon thawing, transferred in a cyclical manner into more appropriate and physiologically relevant conditions [11]. Some reports suggest that pregnancies achieved via FET have lower rates of adverse complications, such as antepartum hemorrhaging, and superior infant outcomes, such as a decreased rates of perinatal death and increased weight at birth [10].

To the best of our knowledge, no studies have investigated the possible relationship between low BMI and neonatal outcomes in FET cycles. Previously published data exclusively focused on fresh IVF cycles, without ruling out the possibility of adverse fetal growth caused by a hyperestrogenic milieu [12]. This study therefore sought to explore how maternal low BMI influences pregnancy and neonatal outcomes in women undergoing FET.

## Methods

### Study subjects

This was a retrospective cohort study conducted between January 2009 and December 2018 at the Department of Assisted Reproduction of the Ninth People’s Hospital of Shanghai Jiao Tong University School of Medicine. Any women during this time scheduled to complete their initial FET cycle were enrolled to participate based on the following inclusion criteria: 1) 20–40 years old; 2) cycle day 3 FSH < 10 IU/l. Patients were excluded if they met the following criteria: 1) Ovarian failure as determined by a lack of antral follicles upon ultrasound assessment, FSH > 10 IU/L; 2) Prior diagnosis of diabetes or hypertension; 3) Recurrent miscarriage or ≥2 spontaneous abortions; 4) Chromosomal abnormalities in either spouse; 5) Endocrine abnormalities (polycystic ovarian syndrome, hyperprolactinemia, abnormal thyroid function, etc.). Certain drugs and diseases that can cause underweight are excluded, including amphetamines, hyperthyroidism, Diabetes, Addison’s disease, Sheehan syndrome, Chronic gastritis, Inflammatory bowel disease, Hepatitis, tuberculosis, malignant tumor and anorexia nervosa. This study was approved by the Ethics Committee (Institutional Review Board) of the Ninth People’s Hospital of Shanghai. And the study was conducted according to the Declaration of Helsinki for medical research and informed consent was obtained. Patient demographic information and cycle parameters were recorded in our medical records system. Patient BMI was determined as follows: BMI = weight/height^2^ (kg/m^2^) during the initial patient consult. Based on these BMI results, patients were divided into three categories based on World Health Organization (WHO) criteria: < 18.5 kg/m^2^ (underweight), 18.5–24.9 kg/m^2^ (normal), 25.0–29.9 kg/m^2^ (overweight). Patients with a BMI> 30 kg/m^2^ were not included in this study of low body weight. The weight and height of the patient was measured in the initial IVF/ICSI cycle. The duration was 6.05±5.21 months from the measurement to the pregnancy occurred.

### Endometrial preparation and frozen embryo transfer

Our center adopts a freeze-all strategy, so patients will not undergo fresh embryo transfer until FET fails more than twice. Detailed descriptions of embryo culture, endometrial preparation, and embryonic transfer have been presented in our previous publications [13]. Embryos were assessed as a means of establishing blastomere numbers and morphology, and in order to determine embryonic disintegration levels based upon Cummins’s criteria [14]. Those embryos deemed to be of high-quality (grade 1 or 2, 8-cell embryos) underwent vitrification in order to freeze them three days following the retrieval of the oocytes. Low quality embryos instead underwent extended culture until reaching a blastocyst stage, and on day 5–6 those morphologically normal blastocysts were also frozen.

### Outcomes

Pregnancy outcomes assessed in this study included rates of implantation, clinical pregnancy, ongoing pregnancy, and live births/transfer cycle. Rates of implantation were determined based upon an ultrasound-mediated quantification of gestational sac numbers relative to numbers of embryos transferred. Clinical and ongoing pregnancy rates were determined on the basis of ultrasound-mediated gestational sac and fetal heartbeat detection at gestational week 7 and 12, respectively. Live births were determined based upon the delivery of at least one live child after gestational week 24. We additionally recorded rates of early/late miscarriage (before gestational week 24), stillbirths, ectopic pregnancies, and terminations as a result of fetal developmental issues. Very preterm birth and preterm birth were defined as delivery before 32 and 37 gestation weeks, respectively. Very small for gestational age and small for gestational age were defined as birth weights <3rd and <10th percentiles in the reference group, respectively. Large for gestational age was defined as birth weights >90th percentiles. Z-score was selected to estimate birth weight adjusted for gestational age and sex. The equation is: Z-score = (x – μ)/σ, in which x is the birth weight of a baby, μ is the mean birth weight for the same gestational age and sex, and σ is the standard deviation for the same gestational age and sex. Birth weight percentiles and Z-scores were depended on birth weight reference percentiles for Chinese [15]. Congenital malformations were identified according to International Classification of Diseases Q codes (Q00–Q99) based on registered conditions and diseases [16].

### Statistical analysis

SPSS v18.0 (SPSS Inc., IL, USA) was used for all analyses. For continuous variables, the normality was tested by the graphical use of histograms and Q-Q plots as well as the Shapiro-Wilk test. If data are normally distributed then they were presented as mean with standard deviation (SD), otherwise they were presented as median (min - max). Continuous variables were compared via one-way ANOVA, while categorical variables were compared via chi-squared tests. Logistic regressions were utilized to determined odds ratios (ORs) and associated 95% confidence intervals (CIs) when comparing data between BMI groups. Models were adjusted for a range of possible confounding variables as follows: maternal age, infertility duration, endometrial thickness, embryo quality, number of embryos transferred, and embryo developmental stage. *P*< 0.05 was the threshold of statistical significance.

## Results

### Patient characteristics

The baseline characteristics of the 8755 women enrolled in this study, divided into three BMI subgroups (underweight, normal weight, overweight), are outlined in Table 1.There were no significant between-group differences in age of embryo transfer, age of oocyte retrieval, duration of infertility, antral follicle number, infertility causes, type of infertility, endometrial thickness on transfer day, basal P level, or fertilization method (*P*> 0.05) (Table 1). As found previously [17, 18], the basal FSH, LH, and E2 levels in the overweight group were significantly lower than those in the underweight and normal weight groups (*P* < 0.05). The use of artificial hormone replacement cycles was more common in the underweight (24.71%) and overweight (25.87%) groups compared with normal weight women (22.04%). The proportion of patients undergoing natural cycles decreased with increasing BMI.

**Table 1:** Baseline characteristics of all FET cycles

Characteristic	Underweight (< 18.5 kg/m^2^ *N*=1315)	Normal weight (18.5–24.9 kg/m^2^ *N*=6230)	Overweight (≥25 kg/m^2^ *N*=1210)	Underweight vs. normal weight	Overweight vs. normal weight	Overweight vs. underweight
Age of embryo transfer (years),mean (SD),	33.63±3.51	33.84±3.79	33.69±3.65	0.065	0.205	0.674
Age of oocyte retrieval (years),mean (SD),	33.12±3.43	33.29±3.85	33.17±3.57	0.138	0.316	0.720
Duration of infertility (years),mean (SD),	3.57±1.28	3.62±1.35	3.69±1.62	0.472	0.352	0.219
BMI of women,mean (SD),	17.53±3.07	21.61±1.73	26.37±1.88	**< 0.001**	**< 0.001**	**< 0.001**
Antral follicle count (*n*),mean (SD),	12.41±5.64	12.72±5.68	12.67±6.07	0.072	0.782	0.265
Infertility causes, *n* (%)				0.291	0.988	0.494
Tubal infertility	790 (60.09%)	3917 (62.87%)	762 (62.98%)			
Male factor infertility	83 (6.32%)	373 (5.98%)	75 (6.18%)			
Unexplained infertiltiy	70 (5.31%)	318 (5.11%)	60 (4.98%)			
Combined*	372 (28.28%)	1622 (26.04%)	313 (25.86%)			
Basal FSH (mIU/mL),median (min-max)	5.77 (0.18–9.99)	5.55 (0.63–9.97)	5.43 (0.43–9.99)	**< 0.001**	**0.008**	**< 0.001**
Basal LH (mIU/mL),median (min-max)	5.02 (0.13–17.96)	4.63 (0.15–18.32)	3.74 (0.42–16.22)	**< 0.001**	**< 0.001**	**< 0.001**
Basal E2 (pg/mL),median (min-max)	35.00 (10.00–86.00)	33.00 (10.00–88.00)	32.00 (10.00–92.00)	**0.017**	0.154	**0.009**
Basal P (ng/mL),median (min-max)	0.30 (0.10–0.90)	0.30 (0.10–0.80)	0.30 (0.10–0.90)	0.991	0.126	0.326
Type of infertility, *n* (%)				0.083	0.295	0.069
Primary	840 (63.89%)	4107 (65.93%)	808 (66.79%)			
Secondary	475 (36.11%)	2123 (34.07%)	402 (33.21%)			
Type of FET cycle, *n* (%)				**0.031**	**< 0.001**	**< 0.001**
HRT	325 (24.71%)	1373 (22.04%)	313 (25.87%)			
Natural cycle	290 (22.05%)	1303 (20.91%)	169 (13.97%)			
Ovarian stimulation	700 (53.24%)	3554 (57.05%)	728 (60.16%)			
Endometrial thickness on transfer day,mean (SD)	10.31±3.32	10.47±2.92	10.44±2.24	0.078	0.686	0.253
Fertilization method, *n* (%)				0.088	0.245	0.112
IVF	838 (63.73%)	3860 (61.96%)	743 (61.41%)			
ICSI	326 (24.79%)	1720 (27.61%)	356 (29.42%)			
Half IVF+half ICSI	151 (11.48%)	650 (10.43%)	111 (9.17%)			

Combined* was defined as two or more infertile causes mentioned above

### Pregnancy outcomes

Comparison of the pregnancy outcomes between the three BMI subgroups are presented in Table 2. The distribution of good quality embryos transfer was comparable between groups (*P*> 0.05). There were also no significant differences in the rates of multiple pregnancy, ectopic pregnancy, intrauterine pregnancy, or live birth between the underweight, normal weight and overweight groups (P> 0.05). However, the implantation (underweight: 33.56% vs. normal weight: 37.26% vs. overweight: 35.48%), clinical pregnancy (underweight: 48.14% vs. normal weight:53.85% vs. overweight: 51.32%), and ongoing pregnancy rates (underweight: 43.04% vs. normal weight: 50.47% vs. overweight: 46.45%) were significantly higher in the normal weight group relative to the underweight group (Table 2). Moreover, the miscarriage rate (underweight: 12.64% vs. normal weight: 10.73% vs. overweight: 13.37%) was significantly lower in normal weight group relative to in the overweight group.

**Table 2:** Reproductive outcomes following transferring blastocyst stage embryo

Characteristics	Underweight (< 18.5 kg/m^2^ *N*=1315)	Normal weight (18.5–24.9 kg/m^2^ *N*=6230)	Overweight (≥25 kg/m^2^ *N*=1210)	Underweight vs. normal weight	Overweight vs. normal weight	Overweight vs. underweight
Number of FET	1315	6230	1210			
Number of viable embryos after thawed	2390	11,440	2193			
Embryo quality				0.296	0.171	0.402
High-quality embryos	2321 (97.11%)	11,135 (97.33%)	2126 (96.95%)			
Low-quality embryos	69 (2.89%)	305 (2.67%)	67 (3.05%)			
Clinical pregnancy rate per transfer,%(n)	48.14%(633/1315)	53.85%(3355/6230)	51.32%(621/1210)	**0.018**	0.193	0.186
Implantation rate,%(n)	33.56%(802/2390)	37.26%(4263/11440)	35.48%(778/2193)	**0.019**	0.145	0.178
Miscarriage rate,%(n)	12.64%(80/633)	10.73%(360/3355)	13.37%(83/621)	0.119	**< 0.001**	0.401
Multiple pregnancy rate,%(*n*)	26.69%(169/633)	27.06%(908/3355)	25.28%(157/621)	0.464	0.257	0.354
Ectopic pregnancy rate,%(*n*)	2.59%(17/654)	2.41%(81/3364)	2.04%(13/637)	0.431	0.352	0.321
Intrauterine and ectopic pregnancy rate,%(*n*)	0.61%(4/654)	0.83%(28/3364)	0.47%(3/637)	0.387	0.251	0.515
Ongoing pregnancy rate,%(*n*)	43.04%(566/1315)	50.47%(3144/6230)	46.45%(562/1210)	**0.002**	0.071	0.152
Live birth rate,%(*n*)	42.05%(553/1315)	45.97%(2864/6230)	43.64%(528/1210)	0.057	0.187	0.319

### Perinatal outcomes

Details of perinatal outcomes such as gestational age, Z-score, weight at birth, and sex are summarized in Table 3. These outcomes were largely similar between these three groups, with the exception of rates of VLBW (< 1500 g) (normal weight: 0.58% vs. overweight: 2.03%), very small for gestational age rates (normal weight:1.31% vs. overweight:3.55%) and VPTD (< 32 weeks) (normal weight: 0.82% vs. overweight: 2.03%), which were significantly higher for overweight group relative to normal weight group. Besides, the rate of cesarean section (under weight:60.91% vs. normal weight: 65.42% vs. overweight: 76.65%) was significantly higher for overweight group relative to other two groups.

**Table 3:** Perinatal outcome stratified by BMI

Birth characteristic	Singleton births			Underweight vs. normal weight	Overweight vs. normal weight	Overweight vs. underweight
	Underweight (< 18.5 kg/m^2^ *N*=399)	Normal weight (18.5–24.9 kg/m^2^ *N*=2062)	Overweight (≥25 kg/m^2^ *N*=394)			
Birth weight(g),%(*n*)						
< 1500 g	0.75%(3/399)	0.58%(12/2062)	2.03%(8/394)	0.451	**0.009**	0.112
1500–2499 g	3.51%(14/399)	3.44%(71/2062)	4.82%(19/394)	0.522	0.129	0.239
≥2500 g	95.74%(382/399)	95.98%(1979/2062)	93.15%(367/394)	0.503	0.368	0.414
Z-score	0.04±0.96	−0.03±1.04	0.02±1.02	0.213	0.381	0.776
Very small for gestational age	1.75%(7/399)	1.31%(27/2062)	3.55%(14/394)	0.486	**0.001**	0.115
Small for gestational age	3.51%(14/399)	3.35%(69/2062)	3.81%(15/394)	0.869	0.645	0.823
Adequate for gestational age	85.96%(343/399)	86.42%(1782/2062)	83.76%(330/394)	0.808	0.163	0.386
Large for gestational age	8.77%(35/399)	8.92%(184/2062)	8.88%(35/394)	0.923	0.980	0.956
Gestation weeks at delivery (weeks),%(*n*)						
< 32	0.75%(3/399)	0.82%(17/2062)	2.03%(8/394)	0.589	**0.038**	0.112
32–36	5.01%(20/399)	5.53%(114/2062)	7.36%(29/394)	0.401	0.113	0.126
≥37	94.24%(376/399)	93.65%(1931/2062)	90.61%(357/394)	0.484	0.354	0.371
Child’s sex, %(*n*)						
Male	54.64%(218/399)	54.51%(1124/2062)	46.45%(183/394)	0.507	0.053	0.104
Female	45.36%(181/399)	45.49%(938/2062)	53.55%(211/394)	0.511	0.085	0.101
Mode of delivery,%(*n*)						
Vaginal	39.09%(156/399)	34.58%(713/2062)	23.35%(92/394)	0.131	0.001	< 0.001
Cesarean section	60.91%(243/399)	65.42%(1349/2062)	76.65%(302/394)	0.223	0.033	0.022

### Congenital defects

Based on International Classification of Diseases criteria, 98 live neonates (1.95%) exhibited congenital defects (Table 4). These defects were evident in 20/707 (2.05%) of infants from the underweight group, 66/3663 (1.80%) from the normal weight group, and 12/662 (1.81%) from the overweight group, with no significant differences between groups (*P* > 0.05). There were also no significant differences when infants were assessed based on singleton/multiple births or sex. The defects observed most frequently were those affecting the circulatory system (underweight: 1.56%; normal weight: 0.98%; overweight: 1.06%). Rates of specific defects or affected systems did also not differ significantly between groups.

**Table 4:** Incidence of birth defects in live-born infants and type of malformations according to the classification from code Q00-Q99 in the international classification of Disease,tenth editon

Characteristics	Underweight (< 18.5 kg/m^2^ *N*=707)	Normal weight (18.5–24.9 kg/m^2^ *N*=3663)	Overweight (≥25 kg/m^2^ *N*=662)	Underweight vs. normal weight	Overweight vs. normal weight	Overweight vs. underweight
Number of birth defects, %(*n*)	20 (2.83%)	66 (1.81%)	12 (1.81%)	0.058	0.541	0.151
Singletons	12/399 (3.01%)	43/2061 (2.09%)	9/394 (2.28%)	0.175	0.462	0.347
Multiples	8/308 (2.59%)	23/1602 (1.44%)	3/268 (1.12%)	0.117	0.478	0.168
Birth defects, by sex,%(*n*)						
Male	13/378 (3.44%)	43/1956 (2.19%)	8/358 (2.23%)	0.114	0.544	0.233
Female	7/329 (2.13%)	23/1707 (1.35%)	4/304 (1.32%)	0.203	0.612	0.324
Detailed birth defects,%(*n*)						
Q00-Q07 nervous system	0	2 (0.05%)	0	0.411	0.393	/
Q10-Q18 eye, ear, face, and neck	2 (0.28%)	3 (0.08%)	0	0.187	0.486	0.525
Q20-Q28 circulatory system	11 (1.56%)	36 (0.98%)	7 (1.06%)	0.129	0.495	0.289
Q30-Q34 respiratory system	0	2 (0.05%)	1 (0.15%)	0.411	0.393	0.734
Q35-Q37 cleft lip and cleft palate	0	4 (0.11%)	0	0.587	0.393	/
Q38-Q45 digestive system	0	2 (0.05%)	0	0.411	0.393	/
Q50-Q56 genital organs	2 (0.10%)	2 (0.05%)	1 (0.15%)	0.126	0.393	0.525
Q60-Q64 urinary system	2 (0.10%)	5 (0.14%)	1 (0.15%)	0.317	0.631	0.525
Q65-Q79 musculoskeletal system	1 (0.14%)	1 (0.03%)	2 (0.31%)	0.298	0.063	0.476
Q80-Q89 other malformations	1 (0.14%)	8 (0.25%)	0	0.559	0.589	0.734
Q90-Q99 chromosomal abnormalities	1 (0.14%)	1 (0.03%)	0	0.298	0.283	0.734

### Logistic regression analysis of BMI-related pregnancy or perinatal outcomes

We next conducted a multivariate logistic regression analysis to identify significant differences in pregnancy/ perinatal outcomes as a function of maternal BMI (Table 5). As shown, clinical pregnancy and ongoing pregnancy were significantly lower in the underweight group relative to the normal weight group. In addition, VPTD, VLBW and very small for gestational age were significantly higher in the overweight group relative to the normal weight group.

**Table 5:** Adjusted ORs of pregnancy and neonatal outcomes

	underweight vs. normal weight	overweight vs. normal weight
	Adjusted Odds Ratio(95% CI)	Adjusted Odds Ratio(95% CI)
Clinical pregnancy	0.75 (0.66–0.94)	0.70 (0.19–2.88)
Miscarriage	1.18 (0.87–1.63)	1.62 (1.21–2.36)
Ongoing pregnancy	0.80 (0.64–0.95)	1.37 (0.55–3.30)
Live-birth	0.63 (0.20–2.29)	0.94 (0.77–1.12)
PTD	0.93 (0.80–1.12)	1.06 (0.92–1.23)
Very PTD	1.09 (0.97–1.24)	1.51 (1.09–2.23)
LBW	1.16 (0.87–1.56)	1.14 (0.95–1.37)
Very LBW	1.08 (0.92–1.33)	1.50 (1.38–1.51)
Small for gestational age	1.11 (0.79–1.60)	1.18 (0.87–1.41)
Very small for gestational age	1.14 (0.91–1.44)	1.63 (1.40–1.78)

*PTD* (preterm delivery:< 37 weeks of gestation), very PTD (very preterm delivery:< 32 weeks of gestation), *LBW* (low birth weight: birth weight < 2500 g);VLBW (very low birth weight: birth weight < 1500 g). Analyses were adjusted for age of embryo transfer, age of oocyte retrieval, infertility duration, endometrial thickness, embryo quality, number of embryos transferred, and embryo developmental stage

## Discussion

This is the first large-scale study assessing the effects of BMI on pregnancy/ perinatal outcomes following FET in a Chinese cohort. Our results reveal that rates of implantation and ongoing/clinical pregnancy were significantly lower when mothers were underweight. When mothers were instead overweight, this was associated with a higher rate of very low birth weight or very preterm delivery. There was also a non-significant trend towards lower live birth rate in both the overweight and underweight groups relative to the normal weight group. There were no other significant effects of maternal BMI on perinatal characteristics or rates of congenital defects. While previous studies have found that obesity can be linked to poor IVF outcomes, our results provide new insight into the negative effects of being underweight on FET outcomes.

Many past assessments of how being underweight influences pregnancy outcomes have produced inconsistent results. For example, Cai et al. found that in a study of over 4000 women undergoing ovarian stimulation via the agonist or antagonist protocols, being underweight was linked to a lower rate of live births (low BMI:50% vs. normal BMI:52.4%, *P*=0.198) but this trend was not significant, whereas abortion rates were significantly increased in those with low BMI relative to those with a normal BMI (13.8% vs. 10.7%, *P* = 0.049) [19]. A separate Danish study of 487 women undergoing frozen or fresh embryo transfer, however, detected no differences in rates of ongoing or ectopic pregnancy, miscarriage, or live births in women who were underweight [20]. A separate study of 332 women without PCOS beginning an initial IVF cycle with the downregulation, antagonist, or microflare protocols observed no effect of low body weight on rates of implantation, clinical pregnancy, or ongoing pregnancy [4]. These previous studies had marked differences in methodology, using fresh or frozen embryos and adjusting for different risk factors, thus potentially explaining these divergent results. Lower fertility rates in underweight women can also be a function of lower levels of leptin [21], which is a hormone mainly produced by fat tissue [22]. The receptor for leptin is known to be present within the secretory endometrium, potentially regulating uterine angiogenesis and implantation [23, 24].

Preterm birth and low birth weight are, in that order, the most prominent causes of death among neonates [25]. In past work assessing outcomes of spontaneous pregnancies among underweight women, results have suggested that lower maternal BMI is linked to higher rates of both of these negative outcomes [26, 27], with similar findings for assessments of women undergoing fresh IVF cycles [18]. This was in contrast to our results, which suggested largely similar perinatal outcomes among women with a lower BMI relative to normal BMI group. Indeed, past work of singleton children born following FET has suggested lower rates of both preterm birth and low birth rate relative to children born as a result of either fresh embryo transfer or spontaneous conception [28]. This, coupled with our results, suggests the possibility that the freezing of embryos may improve outcomes, and that this is particularly true in the case of mothers who are underweight. One possibility explaining these better FET outcomes may be that the freeze-thaw process selects for higher quality embryos, as only these are able to survive this process, and that these higher quality embryos may thereafter be linked to lower rates of LBW and preterm birth [29, 30].

Unlike studies of underweight mothers, overweight mothers were found to have a higher risk of miscarriage, in addition to higher very low birth weight (< 1500 g), very small for gestational age (<3rd percentiles) and very preterm delivery (< 32 weeks) risks. This was consistent with other past studies, which have found being overweight to be linked to reduced fertility [2], and to higher miscarriage rates and obstetric risk [31, 32]. Abnormally high body weight is known to be linked to changes in overall carbohydrate metabolism and increasing resistance to insulin [33], which is often associated with inflammation mediated by interleukin (IL)-1ß, IL-6, and tumor necrosis factor ɑ [34, 35]. Stress as a consequence of physical infections or psychological factors can drive rising plasma levels of contrainsulin hormones such as cortisol or placental growth hormone [33]. Elevated glucose levels in mothers have also been found in some studies to be associated with higher rates of subclinical infections, potentially resulting in higher rates of chorioamnionitis [36]. Such subclinical infection are also linked with systemic inflammation, but can also be asymptomatic and result in instances of VPTD [36].

Among women undergoing FET, we observed no significant variation in congenital malformation rates as a function of BMI, which is largely consistent with work by Best et al. [37], who in a study of spontaneous conception observed no link between BMI and congenital deformity rates with the exception of an increase in cardiovascular malformations in underweight women. Unlike this past study, ours was among the first examining comparable outcomes in the context of a freeze-all-based FET approach. As our data regarding congenital defects came from patient questionnaires, there is a risk that any minor defects may have been overlooked, although this is unlikely to affect the overall study outcomes with respect to infant characteristics at birth.

Our study has several key strengths that lend the results credibility. For one, we had a large sample size and access to largely complete records with respect to the IVF protocols of enrolled patients, as well as detailed information on the resultant pregnancies and outcomes. As these results were collected from a single center, they also have the potential to be associated with a lower rate of heterogeneity than had they been derived from a broader multi-center study.

There are also several important limitations to this work. For one, as the study was retrospective in nature it is important that future randomized controlled trials validates the results discussed herein. Our results regarding overweight mothers was limited to results from a single group (BMI:25.0–29.9), whereas a larger dataset including obese (BMI 30.0–49.9) and superobese (BMI > 50) mothers may have provided additional insights. The weight and height of the patient was measured in the initial IVF/ICSI cycle. It took 6.05±5.21 months from the measurement to the pregnancy occurred, which may affect the results. In addition, while we sought to control for as many confounding variables as possible, some may have unintentionally still introduced bias into our study results. Further research is needed on the effects of pre-pregnancy maternal underweight on pregnancy/ perinatal outcomes following FET.

## Conclusions

In summary, our results indicate that being underweight is linked to certain negative pregnancy/ perinatal outcomes for mothers undergoing FET. There is thus potential value in weight-related counseling not only for overweight women considering IVF, but also for those who are underweight in order to improve outcomes.

## Data Availability

The data used and analyzed during the study are available from the corresponding author if the request is reasonable.

## Abbreviations

BMI: Body mass index
FET: Frozen-thawed embryo transfer
IVF: In vitro fertilization
ART: Assisted reproductive technology
FSH: Follicle stimulating hormone
LH: Luteinizing hormone
E2: Estradiol
OR: Odds ratio
CI: Confidence interval
PTD: Preterm delivery
VPTD: Very preterm delivery
LBW: Low birth weight
VLBW: Very low birthweight
PCOS: Polycystic ovary syndrome
